# Caution: shortcomings of traditional segmentation methods from magnetic resonance imaging brain scans intended for 3-dimensional surface modelling in children with pathology

**DOI:** 10.1007/s00247-023-05692-9

**Published:** 2023-05-30

**Authors:** Anith Chacko, Sean Schoeman, Shyam Sunder B. Venkatakrishna, Samuel Bolton, Andrew I. U. Shearn, Savvas Andronikou

**Affiliations:** 1 CRICBristol, 60 St Michael’s Hill, Faculty of Health Sciences, University of Bristol, Bristol, BS28DX UK; 2 Children’s Hospital of Philadelphia, Philadelphia, PA USA; 3 Perelman School of Medicine, University of Pennsylvania, Philadelphia, PA USA

**Keywords:** Brain, Children, Hypoxic ischemic injury, Magnetic resonance imaging, Printing, Three-dimensional, Segmentation

## Abstract

This technical innovation assesses the adaptability of some common automated segmentation tools on abnormal pediatric magnetic resonance (MR) brain scans. We categorized 35 MR scans by pathologic features: (1) “normal”; (2) “atrophy”; (3) “cavity”; (4) “other.” The following three tools, (1) Computational Anatomy Toolbox version 12 (CAT12); (2) Statistical Parametic Mapping version 12 (SPM12); and (3) MRTool, were tested on each scan—with default and adjusted settings. Success was determined by radiologist consensus on the surface accuracy. Automated segmentation failed in scans demonstrating severe surface brain pathology. Segmentation of the “cavity” group was ineffective, with success rates of 23.1% (CAT12), 69.2% (SPM12) and 46.2% (MRTool), even with refined settings and manual edits. Further investigation is required to improve this workflow and automated segmentation methodology for complex surface pathology.

## Introduction

Accurately printed 3-dimensional (D) models can be used to convey morphological information of the brain and especially the brain surface to non-radiologists [1, 2]. To date, 3-D printing of the brain has focused on adult brains and utilized normal brains or brains with little abnormality. The available literature does not cover the process and problems associated with 3-D printing of pediatric brains, especially with significant pathology such as severe cortical thinning (atrophy) and focal areas of encephalomalacia (parenchymal fluid-filled cysts). These pathologies pose great challenges to 3-D printing of quality models. We aimed to explore the utility and adaptability of automated segmentation methods in the workflow to produce 3-D models of magnetic resonance imaging (MRI) brain scans with accurate representation of surface brain anatomy in children with pathology.

## Description of new technical innovation

This study examined 35 abnormal MR brain scans in children with cerebral palsy due to prior perinatal hypoxic ischemic injury, who underwent delayed MRI. Some patterns of hypoxic ischemic injury only involve portions of the cortex, with related increased complexity of a 3-D mapping workflow to convert MR images into 3-D surface reconstructions. This has previously been documented in adults [3, 4]. The utility of 3-D printing for accurate anatomical representation/biomimicry to facilitate medical teaching, communication with laypeople and legal proceedings is being explored. The 3-D printing workflow is dependent on accurate segmentation of MR scan data. We tested and attempted to improve the segmentation workflow from three common segmentation tools as applied to abnormal pediatric MR brain scans.

**Fig. 1:**
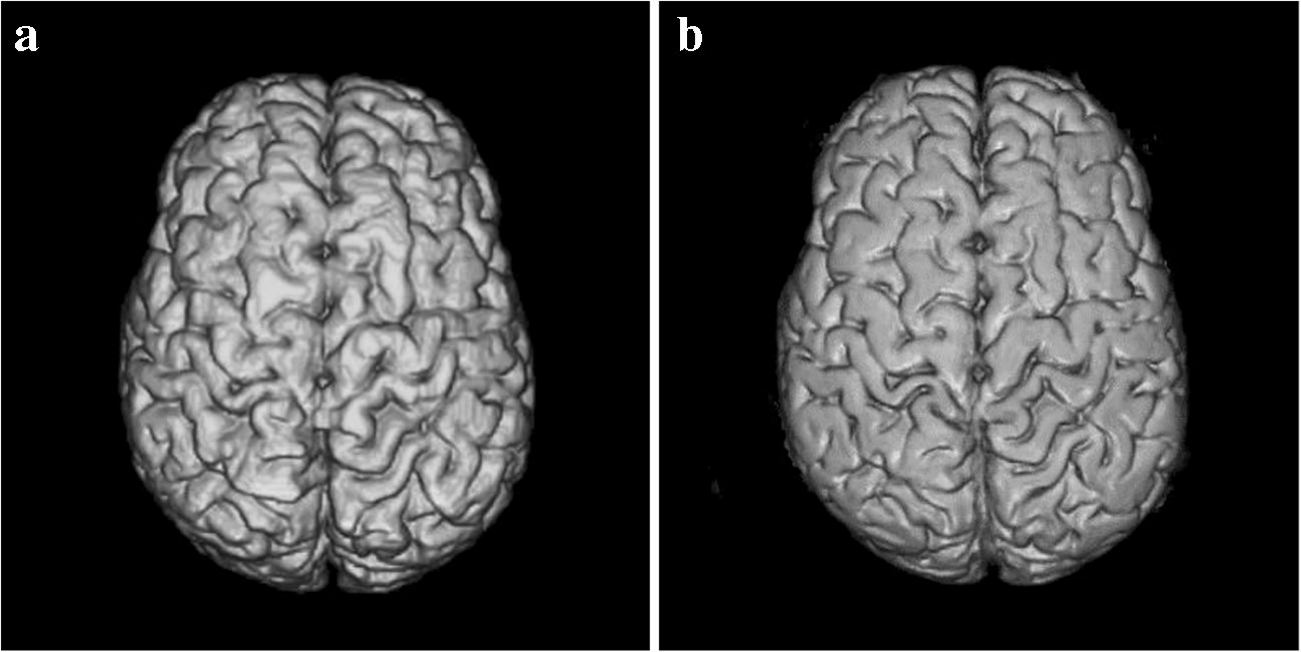
A 5-year-and-8-month-old girl with a normal magnetic resonance imaging brain scan. **a**, **b** Two successful segmentations produced using the Computational Anatomy Toolbox version 12 segmentation method in the 3-dimensional Standard Tessellation Language. The volume model (**a**) is visually higher in quality and demonstrates more cortical gyral and sulcal detail than the volume model (**b**) which shows flattened and less defined gyri. Gyral and sulcal detail is an important component of brain surface demonstration

**Fig. 2:**
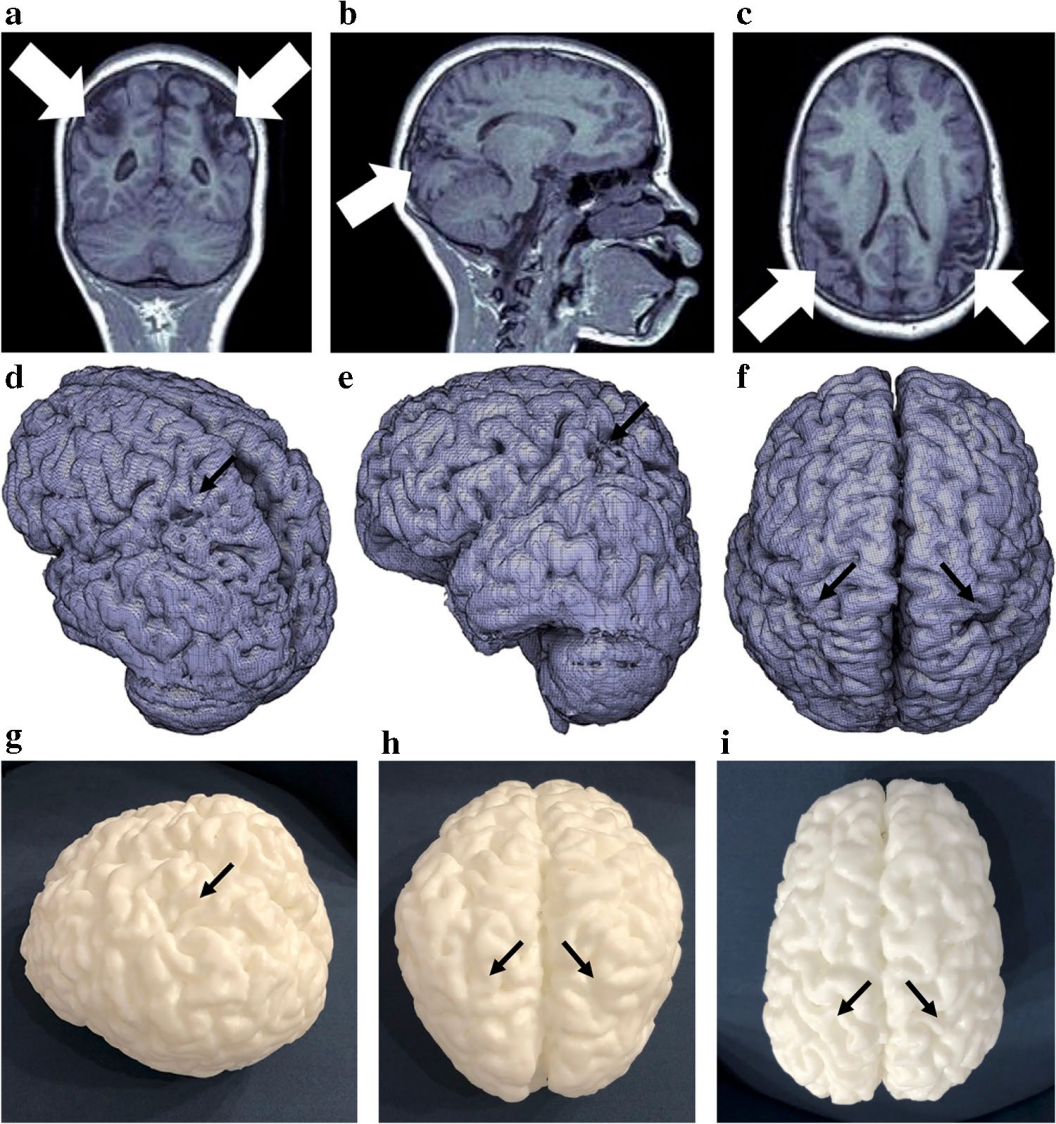
A 10-year-and-11-month-old girl who suffered a perinatal hypoxic ischemic injury with a watershed pattern of injury. **a**–**c** Coronal (**a**), sagittal (**b**) and axial (**c**) T1W magnetic resonance images show bilateral and symmetric cortical and subcortical atrophy in the posterior and perisylvian watershed regions (*arrows*). **d**–**f** Oblique (**d**), lateral (**e**) and vertex (**f**) views of the 3-dimensional (D) mesh virtual model with the corresponding areas of posterior and perisylvian watershed atrophy seen as prominence of the surface sulci with decreased size of the gyri (*arrows*). **g**–**i **Oblique (**g**), posterior vertex (**h**) and center vertex (**i**) photographic views of the corresponding 3-D print models show the affected areas (*arrows*)

**Fig. 3:**
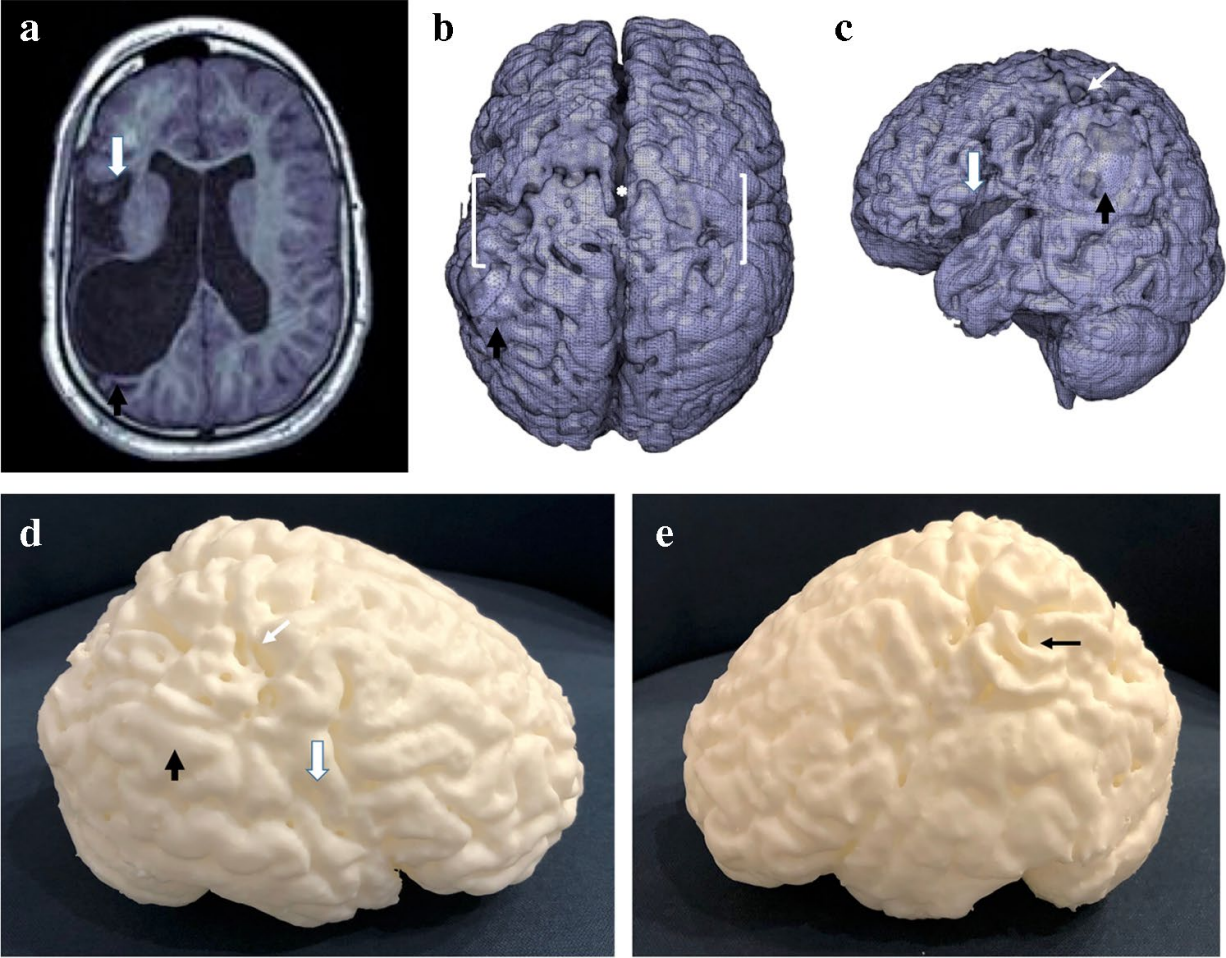
An 11-year-and-6-month-old boy with combined acute-profound and partial-prolonged hypoxic ischemic injury sustained perinatally. **a** Axial T1W magnetic resonance image shows focal asymmetric atrophy with a wide right peri-Sylvian fissure (*white arrow*). There is an expanded posterior body of the right lateral ventricle approximating the surface of the right parietal lobe (due to regional atrophy) and a very thin cortical ribbon (*black arrow*) accurately depicted in the 3-dimensional (D) images. **b**, **c** Vertex (**b**) and right-sided lateral (**c**) views of the 3-D mesh show bilateral asymmetric atrophy of the posterior peri-Sylvian and peri-Rolandic regions (*brackets*). The right is more affected than the left, although with an intact cortical rim, as evidenced by the expanded and smooth cortex overlying the occipital horn of the right lateral ventricle (*black arrow* in **b**). The smooth cortex is also seen in  (**c**)  (*black arrow*). Interhemispheric widening (*asterisk *in **b**) is a sign of atrophy. There is also atrophy of the superior and middle regions of the right peri-Sylvian fissure, which represents the watershed between all three major vessels supplying the brain (*small white arrows*) with associated widening of the peri-Sylvian fissure (*large white arrow* in **c**).  **d**, **e** Right (**d**) and left (**e**) lateral views of the 3-D print model accurately show the corresponding areas of involvement. There is widening of the peri-Rolandic (*small white arrow*) and peri-Sylvian (*large white arrow*) regions. The 3-D model accurately depicts the thin cortical mantle overlying the occipital horn of the right lateral ventricle (*black arrow* in **d**). The area of atrophy in (**e**) at the junction of the peri-Sylvian fissure, medial peri-Rolandic region and posterior intervascular watershed (*arrow*) is in keeping with a combined partial prolonged and acute profound hypoxic ischemic injury

The included image sets were distributed into four categories based on imaging-identified structural abnormalities: “normal”, demonstrating no visible structural brain abnormalities (Fig. 1); “atrophy”, demonstrating general or regional watershed cortical volume loss with or without ulegyria (Fig. 2); “cavity”, demonstrating expanded cystic structures close to the brain surface (focal or multi-cystic encephalomalacia) or significantly expanded ventricles (whether due to ex vacuo dilation or hydrocephalus) (Fig. 3); and “other”, demonstrating non-specific surface brain abnormalities not conforming to the above class descriptions.

**Fig. 4:**
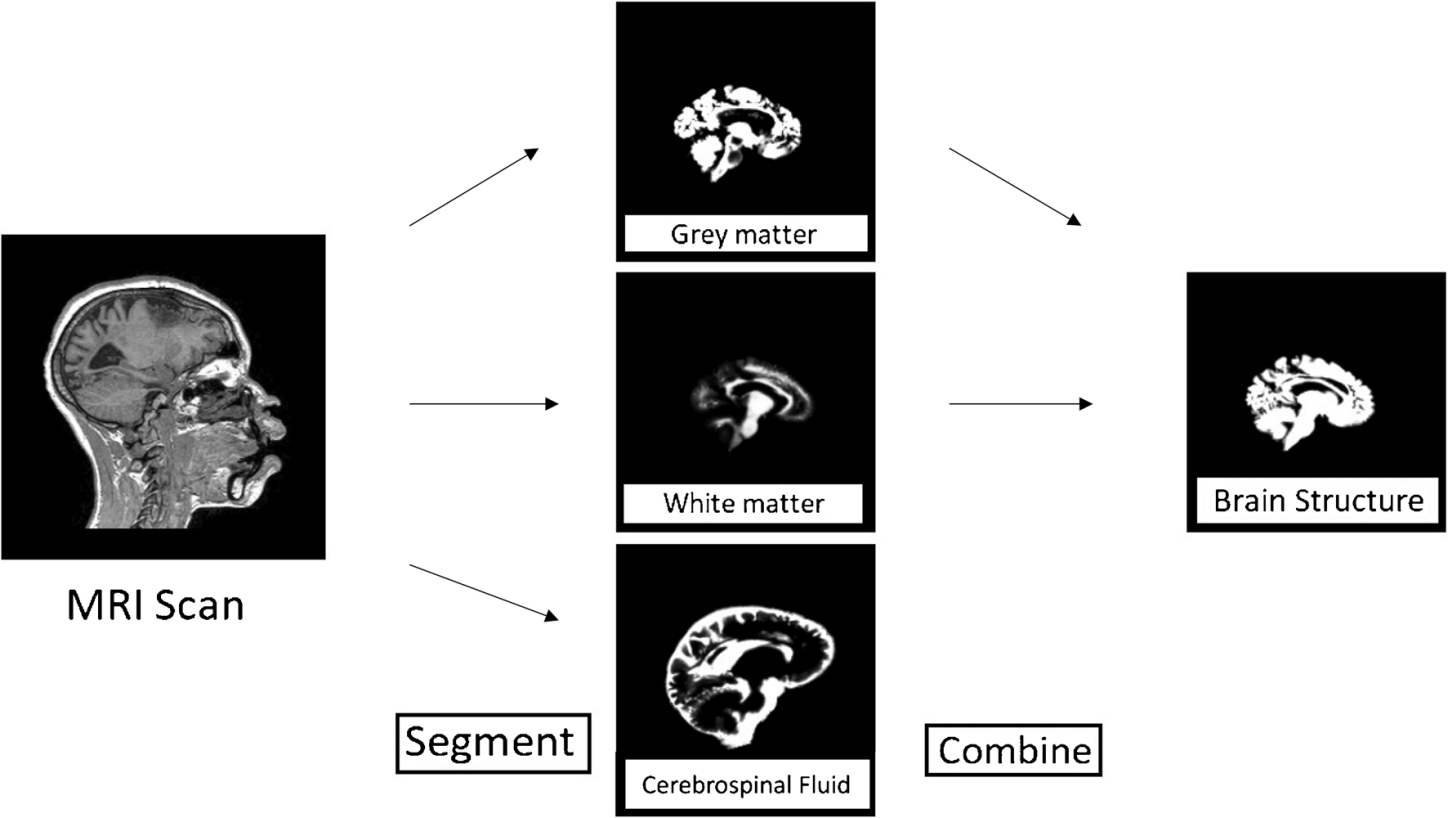
Segmentation into separate tissue classes (gray matter, white matter and cerebrospinal fluid) then combining relevant classes (gray and white matter only) into a volume structure *MRI* magnetic resonance imaging

The process, from scan to 3-D model, has four steps: (1) segmentation; (2) surface meshing; (3) standard tessellation language (STL) file (.stl) preparation; (4) 3-D printing. Digital Imaging and Communications in Medicine (DICOM) image sets of MRI brain scans are acquired and converted to Neuroimaging Informatics Technology Initiative (NIfTI) files—3-D arrays of the original MR scan intensities organized into 1 mm × 1 mm × 1 mm 3-D, voxels. The NIfTI files are segmented; the skull and cerebrospinal fluid are stripped to leave only brain tissue for 3-D modeling (Fig. 4).

Multiple segmentation workflows exist for processing MR images [3, 5]. We used three methods accessible from the MatLab (The MathWorks Inc., Natick, MA) computing environment: Computational Anatomy Toolbox version 12 (CAT12), Statistical Parametric Mapping version 12 (SPM12) and MRTool. CAT12 uses voxel-based morphometry (VBM), while SPM12 and MRTool both employ tissue probability mapping (TPM). We used a custom pediatric probability template from Cincinnati Children’s Hospital combined with standard provided templates [6]. In the VBM analysis technique used by CAT12, the raw image (native space) undergoes dual segmentation of both the gray and white matter. Binarization of the energy assigned to a voxel yields a single tissue class.

After segmentation, the workflow defined a volume based on a surface meshing of the segmented MRI brain. The surface, represented by voxels, is approximated in a standard tessellation language (STL) “mesh” format and the internal volume is effectively discarded. Each component of the mesh is defined by three coordinates and outward-facing normal vectors. There were three methods used to generate the STL surface mesh files; all were accessed via the MatLab computing environment: (1) Iso2surf, (2) Isosurface, (3) MRIcroS.

Meshing errors, though usual, require repair because surface “holes” cause printing errors. The rate of meshing errors acts as a proxy to gauge effectiveness of a segmentation method. Metrics used to analyze the accuracy of STL surface generation include volume of the generated STL file compared to the volume of the corresponding NIfTI file: (1) surface area to volume ratio for each volume generated was compared among the segmentation methods for a given scan and (2) visual assessment comparing STL files with a 3-D representation of the source NIfTI file. For scans not successfully segmented with default settings, the effects of specific default settings were inspected within each method. Manual modification of settings was performed to improve segmentation outcomes as below:

For CAT12:

- *Strength of Inhomogeneity Correction*: Controls the level of bias regularization and bias Gaussian smoothness Full Width at Half Maximum (FWHM) cutoff and measures the strength of inhomogeneity bias correction applied prior to segmentation.
- *Affine Pre-processing (APP)*: Additional pre-processing bias correction aiming to reduce failure in deviating anatomy.
- *Strength of Local Adaptive Segmentation (LAS)*: Without bias, tissue intensity is variable. LAS is a region growing segmentation approach designed to overcome tissue variances and increase correct segmentation [7].
- *Strength of Skull Stripping*: Attempts to isolate the brain by removing the skull before processing, leading to more accurate segmentation.
- *Strength of Final Clean Up:* Attempts to remove meninges and correct for the partial volume effect.

For SPM12 and MRTool:

- *Bias regularization*: Determines the extent to which a non-uniformity correction field should be modelled by the algorithm. Lighter regularization results in a stronger correction field being modelled (hence stronger bias correction).
- *Full Half Width at Maximum Cutoff*: The non-uniformity intensity model used is Gaussian; this option determines the shape of the width of the curve. To model smooth low frequency fields a large cutoff should be used and vice versa.
- *Tissue Probability Map*: Template which represents the probability of a particular tissue type appearing at a particular spatial location—from previous (normal) patients.

## Results

**Fig. 5:**
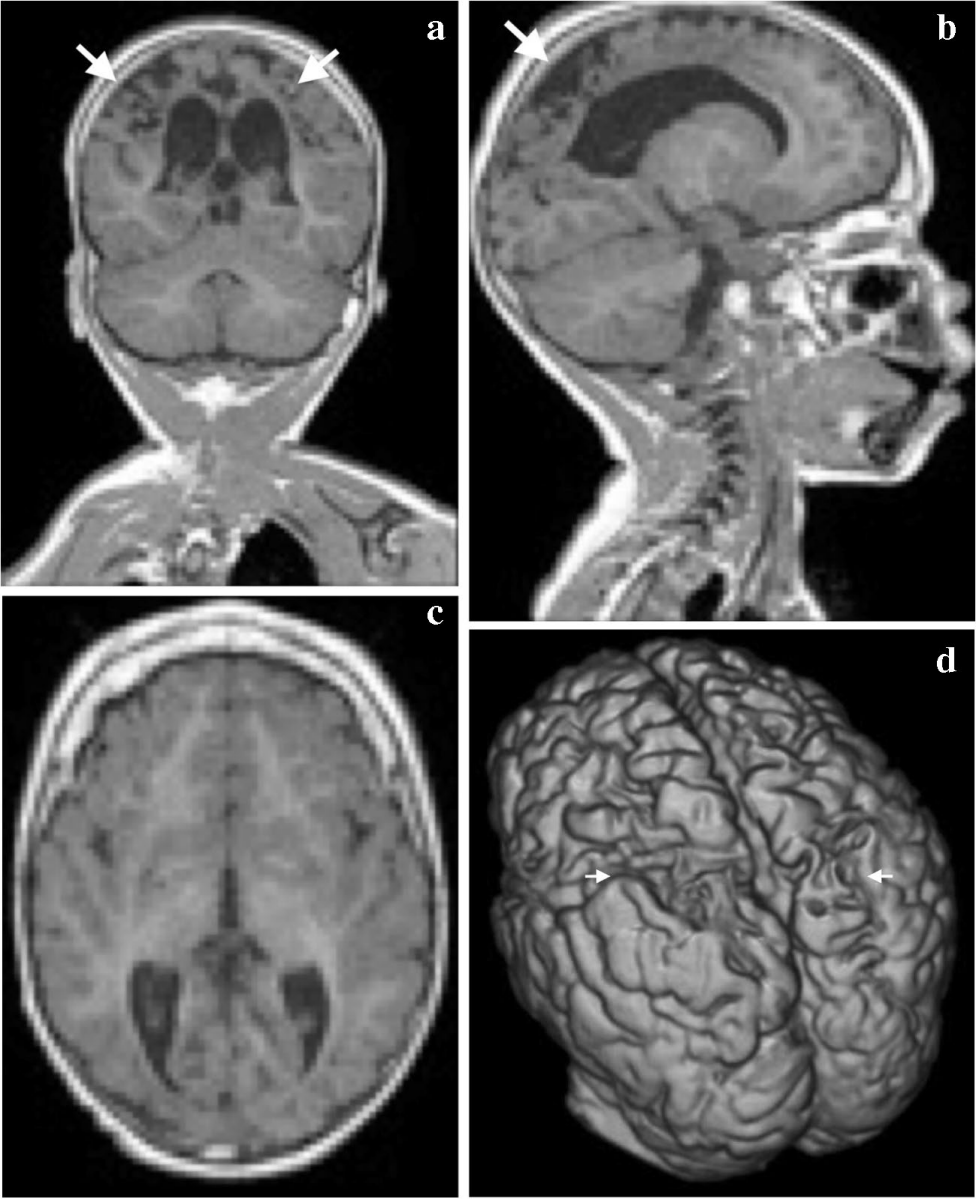
A 2-year-and-2-month-old boy who suffered an acute-profound perinatal hypoxic ischemic injury. **a**–**c** Multiplanar coronal (**a**), sagittal (**b**) and axial (**c**) reconstructions of T1-weighted magnetic resonance images demonstrate bilateral, relatively symmetric peri-Rolandic volume loss (*arrows *in **a** and **b**). **d** High-fidelity three-dimensional surface mesh achieved by generating a standard tesselation language model using the Statistical Parametric Mapping version 12 technique accurately demonstrates the fine cortical detail at the atrophic peri-Rolandic regions (*arrows*)

The described workflow was applied to the 35 image sets with three segmentation methods applied to each. Initially, 22 models were successfully segmented across the three automated methods. This increased to 31 after various optimizations in segmentation. There were 4 MR brain scans, belonging to the “cavity” category, that failed all segmentation methods. The results of manual optimization and modification of default segmentation settings with recommendations (relative to scan class) are outlined in Tables 1 and 2, while Table 3 outlines the comparative success among the three segmentation methods, with respect to MR brain scan class. An example of successful segmentation is shown in Fig. 5.

**Table 1:** Three default settings of Statistical Parametric Mapping version 12 (SPM12) and MRTool segmentation methods and corresponding recommended manual edits according to scan class (presence and type of pathology)

Setting	Modality	Default	Recommendation (normal/other)	Recommendation (cavity/atrophy)
Bias Regularization	SPM12	Light	Extremely light	Extremely light
	MRTool	Very light	Extremely light	Extremely light
Full half-width at maximum	SPM12	60 mm	40 mm	30 mm
	MRTool	60 mm	30 mm	30 mm
Tissue Probability Map	SPM12	Standard	Pediatric	Pediatric
	MRTool	Standard	Pediatric	Pediatric

**Table 2:** Default settings in Computational Anatomy Toolbox version 12 (CAT12) segmentation method and corresponding recommendations for all classes (presence and type of pathology)

Setting in CAT12	Default	Recommended (all)
Strength of Inhomogeneity Correction	Medium	Medium
Affine Pre-processing	Rough	Rough
Strength of Local Adaptive Segmentation	Medium	None
Strength of Skull Striping	Medium	Light
Strength of Final Clean Up	Medium	Light

**Table 3:** Individual and combined success rates of Statistical Parametric Mapping version 12 (SPM12), Computational Anatomy Toolbox version 12 ( CAT12) and MRTool default and manually edited settings across the four classes of magnetic resonance imaging brain scans *N/A* not applicable

	Number of patient scans (image datasets)				
	Normal	Other	Atrophy	Cavity	Total
	12	5	5	13	35
Successful segmentation:					
CAT12					
- Default setting	11 (91.7%)	5 (100%)	2 (40%)	2 (15.4%)	20 (57.1%)
- New settings	11 (91.7%)	5 (100%)	3 (60%)	3 (23.1%)	22 (62.9%)
- New setting + manual edits	N/A	N/A	N/A	N/A	N/A
SPM 12					
- Default setting	10	5 (100%)	4 (80%)	3(23.1%)	22 (62.9%)
- New settings	11 (91.7%)	5 (100%)	5 (100%)	6 (26.2%)	27 (77.1%)
- New setting + manual edits	11 (91.7%)	5 (100%)	5 (100%)	9 (69.2%)	30 (85.7%)
MRTool					
- Default setting	12 (100%)	5 (100%)	3 (60%)	2 (15.4%)	22 (62.9%)
- New settings	12 (100%)	5 (100%)	4 (80%)	4 (30.8%)	25 (71.4%)
- New setting + manual edits	12 (100%)	5 (100%)	4 (80%)	6 (46.2%)	27 (77.1%)

**Fig. 6:**
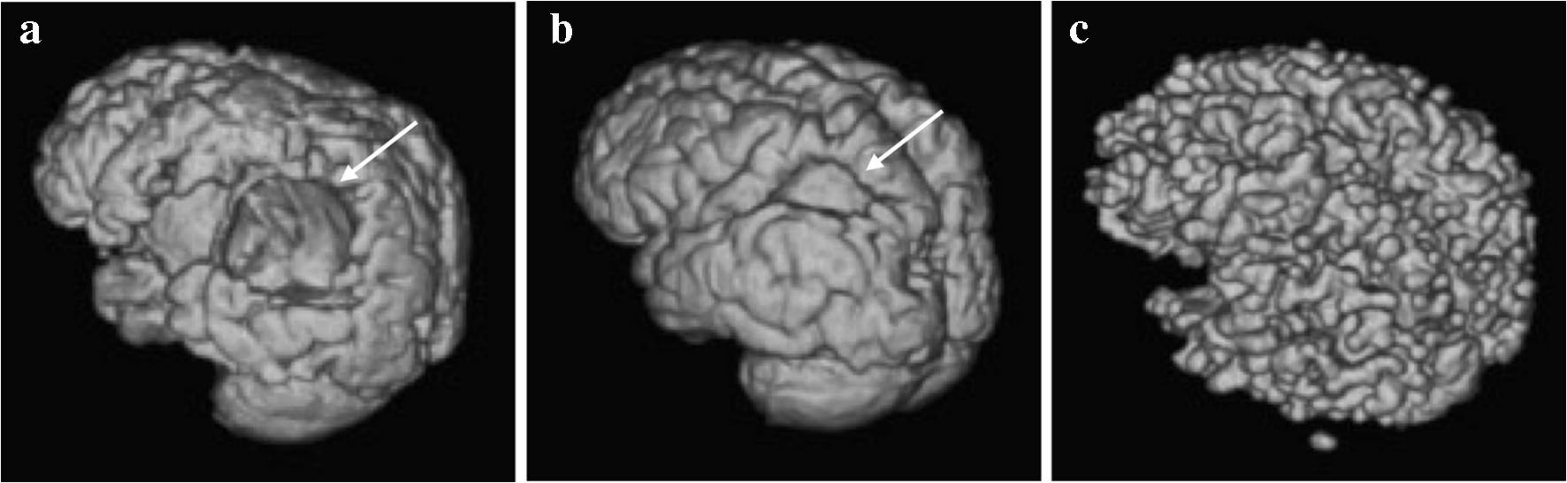
Standard tessellation language surface model examples depicting unsuccessful segmentation of an MRI of the brain in a 5-year-and-2-month-old boy with default settings for (**a**) Computational Anatomy Toolbox version 12, (**b**) Statistical Parametric Mapping version 12, and (**c**) MRTool segmentations. **a** Shows a large open hole in the volume (*arrow*). **b** Compared to (**a**), this image shows a  smaller hole in the volume (*arrow*). **c** There is diffuse exclusion of outer gray matter (incorrect segmentation of the gray matter) in the entire volume, as demonstrated by the extreme irregular sulcal pattern when compared to the models in (**a**) and (**b**)

Automated segmentation via CAT12, SPM12 and MRTool was almost universally successful in the “normal” and “other” classes of MRI brain scans. With default settings, there were success rates of 91.7%, 91.7% and 100%, for CAT12, SPM12 and MRTool, respectively for normal brains, and a 100% success rate for all methods for the “other” class. With respect to the “atrophy” and “cavity” classes, segmentation was less successful. In the “atrophic” group, CAT12 was successful in 60%, SPM12 in 100% and MRTool in 80%. In the “cavity” group, CAT12 was successful in 23.1%, SPM12 in 69.2% and MRTool in 46.2%. Unsuccessful segmentation is demonstrated in Fig. 6. When using default settings across all methods, the maximum success rate for “atrophy” and “cavity” category scans was 7 out of 18 (38.9%). The success rate increased to 14 out of 18 (77.8%) using manual and sequential optimization of all steps involved in segmentation.

## Discussion

Manual segmentation of brain tissue by selecting regions of interest and setting tissue intensity thresholds is ineffective and time intensive [8]. On the other hand, automated segmentation encounters difficulty when tissues are non-uniform or pathologic. Non-uniform bias fields cause large variations in intensity for the same class of tissue. Also, as voxels (tetrahedral pixel elements) are of fixed resolution, problems occur when the native resolution from the MR scan (DICOM file) results in the presence of two tissue classes in one voxel, i.e., partial volume effect [9]. Recorded tissue intensities merge and classification errors occur through the binarization of a voxel. Complex segmentation methods mitigate this. One quantitative comparison in 2014 found SPM8 (current version SPM12) the most accurate of the segmentation methods [8].

VBM techniques have been effective in detecting gray and white matter changes in adults suffering from motor neuron disease and in detecting levels of atrophic tissue in the brain [10]. Comparisons among segmentation methods for adults with multiple sclerosis concluded that there is no current best segmentation method for detecting lesions with varying characteristics and scan quality [11]. The above studies focused on detection and diagnosis. Our comparison prioritized each methods’ suitability for producing a 3-D surface model. We showed that in MR scans of children with atrophy or cavities close to the brain surface, segmentation was unsuccessful using previously recommended segmentation methods in default settings.

All three methods used for segmentation (CAT12, SPM12 and MRTool) adequately segmented nearly all the scans in the “normal” and “other” categories, albeit with different degrees of quality. The CAT12 method had a relatively low success rate with “cavity” and “atrophy” category images. Both SPM12 and MRTool methods were more successful with the “atrophy” and “cavity” categories. The successful segmentation rate was increased in both these methods by lowering the bias regularization setting (increasing bias correction). The segmentation volumes were also improved in both by using the pediatric TPM rather than the provided TPM [6]. The “atrophy” category scans were challenging to segment using default settings, but all produced usable models with modified settings. The “cavity” category patients remained the most challenging—three failed segmentations despite modification of various settings, likely due to very thin overlying cerebral mantle resulting in defects in the surface.

**Fig. 7:**
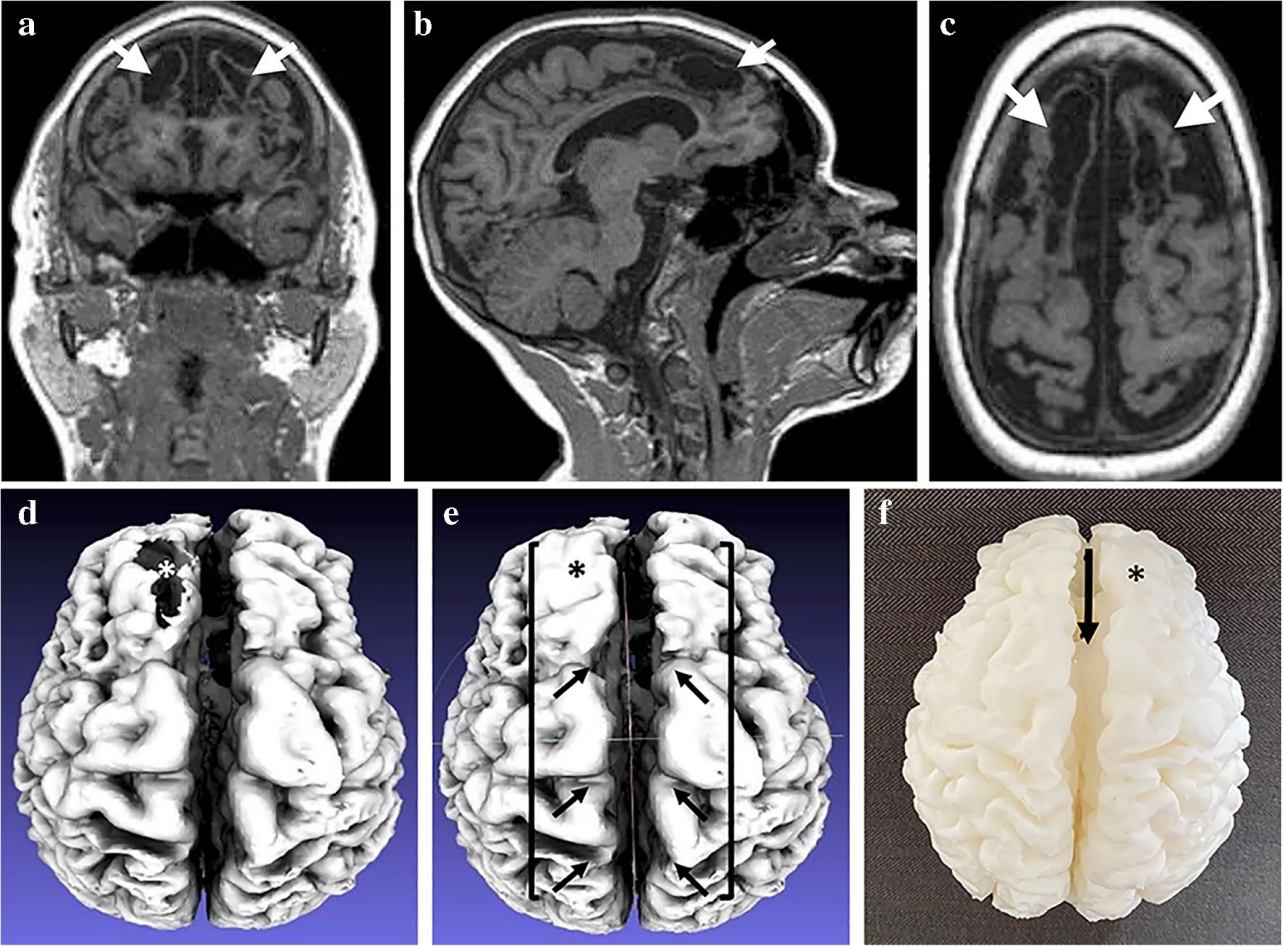
A 10-year-and-5-month-old girl with a previous global partial-prolonged hypoxic ischemic injury affecting the parasagittal watershed regions. **a**–**c** Multiplanar coronal (**a**), sagittal (**b**) and axial (**c**) reconstructions of T1 weighted magnetic resonance images demonstrate bilateral parasagittal cystic encephalomalacia (*arrows*) and very thin residual cortex of the superior frontal lobes. **d** Vertex view of an initial standard tessellation language (STL) surface model which was unsuccessful in demonstrating the thin cortical mantle of the right frontal lobe, leaving a large hole with a view into the cystic cavity involving the right frontal lobe white matter (*asterisk*). **e** Vertex view of a subsequent usable STL surface model created by patching the hole using manual editing (*asterisk*). Significant parasagittal watershed (between *brackets*) atrophy is accurately demonstrated (*arrows*). **f** Vertex view of the final 3-D print model shows the interhemispheric widening (*arrow*) and the area that was patched within the right frontal lobe (*asterisk*)

The efficiency benefits of automated segmentation were lost because various settings for each method required manual adjustment and even this did not guarantee successful segmentation. The difficulties in segmenting the “cavity” class of patients are highlighted in Fig. 7. Automation becomes redundant when a large amount of manual editing is required to build a successful mesh for 3-D surface display. Our comparison of segmentation methods demonstrates problems when using current segmentation methods for surface 3-D model creation in children with pathology. Automated segmentation default settings are designed for adult-type tissue, rendering TPMs ineffective when tissue deviates from normal.

## Conclusion

When proceeding through the workflow to produce a 3-D model of the surface from pediatric brain MRI, investigators should consider the feasibility of segmentation, weighing this up against the ultimate utility of an accurate 3-D model. We have shown that segmentation is unsuccessful when using recommended segmentation methods in default settings for MRI scans in children with atrophy of the cerebral gyri or cavities close to the surface of the brain.

## Data Availability

The datasets generated and/or analyzed during the current study are available from the corresponding author on reasonable request.
